# Development and Validation of a Rule-Based Natural Language Processing Algorithm to Identify Falls in Inpatient Records of Older Adults: Retrospective Analysis

**DOI:** 10.2196/65195

**Published:** 2025-07-08

**Authors:** Xing Xing Qian, Pui Hing Chau, Daniel Y T Fong, Mandy Ho, Jean Woo

**Affiliations:** 1 School of Nursing, Li Ka Shing Faculty of Medicine, The University of Hong Kong, 5/F, Academic Building, 3 Sassoon Road, Pokfulam, Hong Kong, China (Hong Kong), 852 3917 6626; 2 Department of Medicine and Therapeutics, Faculty of Medicine, The Chinese University of Hong Kong, Hong Kong, China (Hong Kong)

**Keywords:** fall-related admissions, electronic medical records, text mining, case detection, natural language processing

## Abstract

**Background:**

In order to address fall underestimation by the *International Classification of Diseases* (*ICD*) in clinical settings, information from clinical notes could be incorporated via natural language processing (NLP) as a possible solution. However, its application to inpatient notes has not been fully investigated.

**Objective:**

This study aims to develop and validate a rule-based NLP algorithm to identify falls based on inpatient admission notes from older patients.

**Methods:**

This retrospective study used 12-year electronic inpatient records of patients aged ≥65 years from public hospitals in Hong Kong. A random sample of 1000 patients was drawn to develop the NLP algorithm. Manual review was the gold standard for assessing the algorithm’s performance, with sensitivity, specificity, precision, and *F*_1_-score calculated at the record, episode, and patient levels. In addition, the study compared the number of falls identified by *ICD* codes and clinical notes independently and combined.

**Results:**

Our rule-based NLP algorithm showed excellent performance, with a sensitivity, specificity, precision, and *F*_1_-score of 93.3%, 99.0%, 87.5%, and 0.903 at the record and episode levels, and 92.9%, 98.3%, 89.7%, and 0.912 at the patient level. The combined identification strategy using *ICD* codes and the NLP method provided the most comprehensive capture of fall-related episodes and fallers.

**Conclusions:**

The NLP method proved efficient and accurate in detecting falls from clinical notes in inpatient episodes. For comprehensive capture of fall episodes and fallers, we recommend the combined use of the NLP algorithm and *ICD* codes, which should be applied in future fall epidemiology studies and clinical practice for identifying high-risk groups of fall interventions.

## Introduction

Falls are a global public concern and impose considerable health care and economic burdens on the older population [1]. The Decade of Healthy Aging Report from World Health Organization (WHO) highlights fall prevention as an urgent health priority [2]. Fall risk screening is inevitable when identifying vulnerable older individuals for assessment and preventive intervention [3,4]. The history of falls has been widely recognized as a vital component in risk screening [3], given that it doubles the fall risk for older adults [5,6].

In the longitudinal studies on the older population, falls were mainly identified through medical (electronic and nonelectronic) or nonmedical records (eg, retrospective telephone or face-to-face interviews and prospective fall diary or calendar) [6,7]. Compared to nonmedical methods, which are subject to withdrawal or loss of follow-up from participants, medical records are more efficient and cost-saving. Hence, hospitals that provide care for fall-related injuries become the ideal sites to identify and track fallers for interventions. Most studies conducted in clinical settings used the *International Classification of Diseases* (*ICD*) codes to detect falls in the electronic medical records (EMRs) of older adults [6]. Nevertheless, the identification based on the *ICD* codes alone may not completely capture the reported falls. Published studies found that the *International Classification of Diseases, Ninth Revision, Clinical Modification* (*ICD-9-CM*) codes missed 8% to 20% of falls in patients [8,9]. Involving supplemental records could increase accuracy for fall identification, in which the clinical note review captures the greatest number of events [10]. The traditional manual note review that requires health care professionals as the reviewer is labor-intensive and time-consuming due to the large volume of records. While a relatively expedient and cost-effective approach is searching for the keyword “fall” in the clinical notes, it is important to note that a simple keyword search alone is insufficient to accurately distinguish between fall-related notes and those that do not. This is because notes without actual falls may also contain the term “fall”, such as “references to high fall risk,” “fall precaution,” or “falling asleep,” which can result in false positives. To enhance accuracy and overcome this limitation, a novel computer-based approach named natural language processing (NLP) has been applied as an effective solution [11].

NLP algorithms are generally categorized into two approaches: rule based and machine learning (ML) based [11]. While both have their merits, rule-based NLP is selected for this study for several reasons. First, rule-based systems rely on explicitly defined linguistic rules, making them highly interpretable and transparent [12]. This allows developers and users to easily understand and modify the rules, ensuring precise performance. Second, rule-based NLP does not require large annotated datasets, unlike ML-based approaches, which demand extensive training data and computational resource [13]. This makes rule-based systems practical for real-time applications or low-resource environments. Third, rule-based systems efficiently handle edge cases and specific linguistic nuances by directly tailoring rules to unique exceptions—such as distinguishing between actual falls and terms like “falling asleep” in clinical notes—without extensive data or training [12]. While ML-based NLP excels with large-scale datasets and broad contexts [13], it often requires more effort to match the precision and domain-specific adaptability of rule-based systems. In recent years, transformer-based pretrained models have emerged as a powerful ML approach, advancing NLP by capturing long-range dependencies and contextual information in text, even with limited annotated data [14]. Due to their ability to model complex linguistic patterns and contextual relationships, transformer-based models may outperform rule-based systems. Nevertheless, these models still require substantial computational resources, which can be mitigated through cloud-based application programming interfaces (APIs) [15]. In health care systems where patient data confidentiality is strictly restricted, the reliance on internet connectivity to use such models poses a significant challenge. Therefore, for local applications, rule-based approaches remain a pragmatic choice.

However, research on rule-based NLP algorithms for fall identification in clinical notes remains limited. Previous studies have developed rule-based NLP algorithms for inpatient, emergency department (ED), and homecare visit notes [16-18], with the inpatient study specifically focusing on Japanese clinical notes [16]. Although the English-language NLP algorithms for ED and homecare visit notes demonstrated satisfactory performance compared to manual review [17,18], these methods may not be directly transferable to inpatient settings. Hence, more studies are warranted to develop specialized rule-based NLP methods for accurate fall identification in inpatient clinical notes. Furthermore, existing studies primarily compared identification strategies based on single record sources of *ICD* codes or clinical notes [8,9]. There is a lack of studies investigating how the combination of these strategies can enhance fall identification. Therefore, to address these gaps, this study aimed to (1) develop and validate a rule-based NLP algorithm to identify falls based on inpatient admission notes and (2) compare the number of falls identified by independent or combined strategies of *ICD* codes and the NLP method.

## Methods

### Study Design and Data Source

We performed a retrospective, territory-wide, cohort study using the 12-year (2007-2018) electronic inpatient records of older adults. The dataset used in this study encompasses admissions from all 43 public hospitals of the Hospital Authority (HA), which manage approximately 80% of hospital admissions in Hong Kong and account for nearly 90% of total bed-days [19]. The data were extracted from the Hospital Authority Data Collaboration Lab (HADCL), and the details were described elsewhere [20]. The clinical records were written in English.

### Sampling

The sampling frame was a cohort of older adults who (1) resided in Hong Kong, (2) were discharged as an inpatient from public hospitals between January 2007 and December 2017, (3) were aged ≥65 years at admission, (4) had a hospital stay of at least one day for their first inpatient episode, (5) were discharged alive, (6) did not have a hospital discharge from public hospitals 365 days before the first inpatient episode, and (7) were not admitted from the nursing home at their first inpatient episode. More details of the criteria are described elsewhere [20].

To ensure adequate statistical power for developing an NLP algorithm, a sample size of at least 500 is recommended [21]. In this study, a sample of 1000 patients was drawn by random sampling. The electronic clinical notes and diagnosis codes of all inpatient admission episodes from selected patients were extracted for analysis. The dataset contains two types of notes: clinical notes and discharge notes. Only the clinical notes, comprising all textual records associated with linked episodes, were used in this study.

### Manual Review

The manual review was considered a gold standard, as consistent with previous studies [16-18]. A total of 2 reviewers independently reviewed each extracted clinical note and determined the corresponding record as being fall related or not, using binary classification. According to the WHO, a fall is defined as an event in which a person unintentionally comes to rest on the ground, floor, or another lower level [1]. An individual who has experienced at least one fall episode is classified as a faller. Reviewers were trained to annotate the clinical notes as fall related if the scenario presented in the notes meet the WHO definition of falls or have specific indicators (eg, “admitted for fall,” “presented with fall,” “fell onto ground,” “slipped and fell,” or “fell when...”). Reviewers relied on these predefined indicators to ensure consistency in their assessments. Any disagreement was resolved by a joint evaluation involving the 2 reviewers and a senior investigator. As each patient could have multiple inpatient admission episodes during the study period and each episode could include multiple records, fall identification was performed at levels of record, episode, and patient. Patients were classified as fallers and episodes were designated as fall related if any associated episodes or records contained falls.

### Development and Validation of the Rule-Based NLP Algorithm

**Figure 1.**
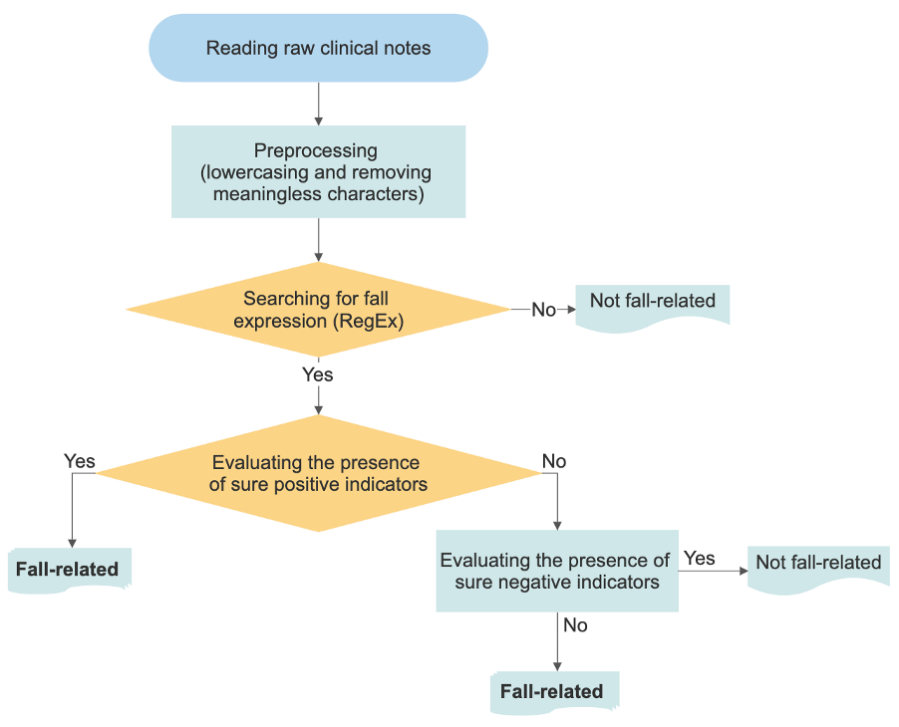
Process of the rule-based natural language processing algorithm development. RegEx: Regular Expression.

Patients were randomly split into the training set for NLP algorithm development and the testing set for validation at a ratio of 80:20 [22]. The development process in the training set included 5 steps (Figure 1). First, clinical notes were preprocessed by lowercasing characters and removing meaningless “Newline Characters” (indicates starting from the next line). Second, the fall expression within the clinical notes was searched by the Regular Expression (RegEx; Section S1 in Multimedia Appendix 1). After filtering out clinical notes with fall expression, the third step evaluated the presence of sure positive indicators (Section S1 in Multimedia Appendix 2) that a fall occurred within the filtered notes. Similarly, the fourth step was evaluating the presence of sure negative indicators (Section S1 in Multimedia Appendix 2) that a fall had not occurred. The sure positive and sure negative indicators were iteratively refined through performance evaluation and rule adjustment. At each cycle, common areas of false positives and negatives were addressed to extend the criteria until the value of sensitivity, specificity, and precision exceeded 90%, and the value of *F*_1_-score exceeded 0.9 at one of the levels for record, episode, or patient [17]. Finally, as fall expression can appear more than once within the same clinical note, the sure positive and negative indicators could coexist in one note. Therefore, clinical notes that (1) had fall expression and (2) had sure positive indicator or did not have sure negative indicator were marked as fall related. Subsequently, the rule-based NLP algorithm was applied to identify falls in clinical notes from the testing set for validation.

### Comparison of Different Identification Strategies

The number of falls identified by the following methods were compared: (1) manual review of clinical notes, (2) NLP algorithm for clinical notes, (3) *ICD* codes, (4) manual review and *ICD* codes, and (5) NLP algorithm and *ICD* codes. Regarding the strategy of *ICD* codes, a fall was identified by the *ICD-9-CM* E880-E888 for accidental falls or 800‐909.2, 909.4, 909.9, 910‐924, 950‐957, 959 for injury (excluding E000, E800-807, E810-838, E840-858, E860-879, E890-999 for specific external causes of injury) [23,24]. Since not all episodes contain both *ICD* codes and clinical notes—some may lack notes while others may lack *ICD* codes, comprehensive fall identification should consider both data sources. Therefore, the combined strategy, incorporating manual notes review and *ICD* codes, was considered the identification standard to compare the remaining strategies. For combined strategies (4) and (5), the record was identified as fall related when either strategy captured a fall. The comparison was restricted to these 1000 patients, as one of the involved strategies was manual review, which could be time-consuming and labor-intensive if extended to the whole dataset.

### Statistical Analysis

The mean and SD values were used to describe the length of clinical notes (number of characters). The falls identified by the developed NLP algorithm were compared with the manual review by confusion matrix to generate standard performance measures of sensitivity, specificity, precision, and *F*_1_-score at the levels of record, episode, and patient in the training and testing sets [25]. Sensitivity refers to the proportion of correctly labeled falls by the NLP algorithm to all falls identified by manual review. Specificity computes the proportion of correctly labeled nonfalls by the NLP algorithm to all nonfalls identified by manual review. Precision reflects the proportion of the correctly labeled falls by the NLP algorithm to all labeled falls by NLP algorithm. *F*_1_-score is the harmonic mean of the precision and sensitivity, with the best value being 1 and the worst being 0. An adequate performance is considered when the value exceeds 90% for sensitivity, specificity, and precision and 0.9 for *F*_1_-score [17]. All data analyses were performed using Python 3.7.6 (CreateSpace).

### Ethical Considerations

This study was approved by the Institutional Review Board of the University of Hong Kong and the Hong Kong Hospital Authority West Cluster (approval UW 23‐046). Informed consent was waived by the Institutional Review Board due to the retrospective, observational nature of the study and the exclusive use of deidentified participant data. Participants were not compensated for their involvement in this study.

## Results

In total, 2153 clinical notes (records) of 2095 inpatient admission episodes from 1000 sampled patients were included in the analysis. The mean length of notes was 1072 characters, with an SD of 916. To develop the NLP algorithm, 800 patients (1720 records and 1672 episodes) were randomly assigned to the training set and the remaining 200 patients (433 records and 423 episodes) were assigned to the testing set.

Regarding the training set for development, the manual review identified 140 fall-related records, 135 fall-related episodes, and 127 fallers. In comparison, the NLP algorithm identified 145 fall-related records, 140 fall-related episodes, and 124 fallers. In the testing set for validation, the manual review identified 30 fall-related records, 30 fall-related episodes, and 28 fallers, while the NLP algorithm identified 32 fall-related records, 32 fall-related episodes, and 29 fallers. As shown in Table 1, falls identified by the NLP algorithm were largely concordant with those identified by manual review at record, episode, and patient levels. Performance metrics of the NLP algorithm were satisfactory for both training and testing sets at all 3 levels (Table 2). In the testing set, the NLP algorithm achieved a sensitivity of 93.3%, a specificity of 99.0%, a precision of 87.5%, and an *F*_1_-score of 0.903 at both the record and episode levels. These measures at patient level were 92.9% for sensitivity, 98.3% for specificity, 89.7% for precision, and 0.912 for *F*_1_-score.

Table 3 presents the comparison of different fall identification strategies, with regards to the number of falls identified at the record, episode, and patient levels. Overall, as compared to identification standard of manual notes review combined with *ICD* codes, the NLP algorithm combined with *ICD* codes identified the highest number of falls across all 3 levels (99.4%-104.3%), followed by the NLP algorithm alone (91.1%-93.5%). Specifically, compared to the identification standard, the NLP algorithm combined with *ICD* codes slightly overestimated the numbers of fall-related records and episodes (~4%) yet underestimated the number of fallers (~1%). In contrast, *ICD* codes alone identified the least number of falls (58.7%-60.7%), as 44 inpatient records did not have *ICD-9-CM* codes.

**Table 1.** Comparison of fall identification by manual review and the rule-based NLP^a^ algorithm.

NLP algorithm and level	Manual review			
	Training set		Testing set	
	Fall, n	Without fall, n	Fall, n	Without fall, n
Record				
Fall	129	16	28	4
Without fall	11	1564	2	399
Episode				
Fall	124	16	28	4
Without fall	11	1521	2	389
Patient				
Fall	116	8	26	3
Without fall	11	665	2	169

a NLP: natural language processing.

**Table 2.** Performance metrics of the rule-based natural language processing algorithm.

Metric	Training set			Testing set		
	Record level	Episode level	Patient level	Record level	Episode level	Patient level
Sensitivity	92.1%	91.9%	91.3%	93.3%	93.3%	92.9%
Specificity	99%	99%	98.8%	99%	99%	98.3%
Precision	89%	88.6%	93.5%	87.5%	87.5%	89.7%
*F*_1_-score	0.905	0.902	0.924	0.903	0.903	0.912

**Table 3.** Number of falls identified by different strategies.

Strategy	Number of falls identified (% of identification standard)		
	Record level	Episode level	Patient level
Manual review and *ICD*^a^ codes^b^	193 (100)	184 (100)	168 (100)
NLP^c^ algorithm and *ICD* codes	201 (104.1)	192 (104.3)	167 (99.4)
Manual review of clinical notes	170 (88.1)	165 (89.7)	155 (92.3)
NLP algorithm for clinical notes	177 (91.7)	172 (93.5)	153 (91.1)
*ICD* codes^d^	114 (59.1)	108 (58.7)	102 (60.7)

a *ICD*: *International Classification of Diseases*.

b Identification standard for comparison.

c NLP: natural language processing.

d In the whole set, 44 records were without *ICD* codes.

## Discussion

### Principal Findings

This study developed a rule-based NLP algorithm to identify falls in clinical notes of inpatient admission episodes based on a random, territory-wide sample of older patients, which achieved excellent performance when compared with manual notes review in the testing set. We also compared different fall identification strategies, including the use of *ICD* codes or clinical notes alone or in combination. Our findings provide a feasible and efficient solution for fall screening and shed light on future research and practice related to this field in the clinical setting.

Using NLP algorithms to identify falls can significantly reduce the time and human resources required for the manual notes review. Previous studies have developed NLP methods for fall identification in clinical notes of inpatient wards [16,26,27], and the performance of NLP methods varied. Toyabe [16] derived a rule-based NLP algorithm to detect falls in Japanese progress notes and achieved a sensitivity of 100.0% and a specificity of 98.4%, but with a low precision (6.2%) and an *F*_1_-score of 0.118 at the record level. Shiner et al [26] developed an ML-based NLP method to capture falls in English progress notes, achieving a relatively low sensitivity of 44.1%, a specificity of 97.0%, a precision of 67.2%, and an *F*_1_-score of 0.533 at the record level. Nakatani et al [27] also developed an ML-based NLP algorithm to differentiate fallers and nonfallers in Japanese nursing notes, with a moderate sensitivity of 76.9% and a specificity of 78.5% at the patient level. In this study’s testing set, the sensitivity, specificity, precision, and *F*_1_-score of the rule-based NLP algorithm were 93.3%, 99.0%, 87.5%, and 0.903 at the record level and 92.9%, 98.3%, 89.7%, and 0.912 at the patient level, respectively. The values of these measures were generally higher than previous studies, indicating the promising performance of our rule-based NLP algorithm. Notably, our NLP method was rule based and could be easily applied by researchers or health care professionals with limited programming knowledge to facilitate fall identification in inpatient settings. The rule-based NLP algorithm is also flexible for adjustment based on the different patterns of clinical notes and could be generalized to different systems, such as the ED clinical notes. In addition, future studies may explore the extended application of the NLP method in extracting other fall-related information, such as the circumstance of falls, the mechanism of falls, and resulting injuries, which are important in informing the prognosis and required interventions for patients who have experienced falls [3].

In the epidemiological studies for falls in older adults, the number of fall episodes and fallers are typically the outcomes of interest in estimating the risk (eg, incidence and proportions) and associations. Our findings suggest that the fall identification strategy involving *ICD* codes and clinical notes provides the most comprehensive capture of fall-related episodes and fallers. Identification of falls based on *ICD* codes alone is known to underestimate the number of falls [8,9,28]. Incorporating information from clinical notes is especially beneficial in addressing this issue. Given the limitations of manual review, the NLP algorithm offers an efficient way for applying the combined strategies of clinical notes and *ICD* codes in fall identification on a large scale. Our study found that compared with the combination of manual review and *ICD* codes, the combination of NLP algorithm and *ICD* codes slightly overestimated the number of fall-related episodes (~4%) but not the number of fallers. Therefore, the fall identification strategy combining NLP algorithm and *ICD* codes with feasibility and accuracy has promising application value in risk screening and patient tracking.

The health care workforce shortage is a global problem. Identification of efficient methods to enhance the quality of patient care and safety is a priority agenda in health care field. In terms of implications to practice, there are posthospital health care services or fall prevention programs designed to improve functions and reduce falls for patients recently discharged from hospitals. Fall history is a dominant risk factor for posthospital falls in older patients [6]. Since hospitals are ideal sites for identifying and tracking patients with a history of falls, this combined strategy of NLP method and *ICD* codes is expected to benefit posthospital fall interventions both at baseline and during follow-up. The combined strategy allows for standardized and consistent screening across an extensive EMR dataset, and it can be integrated into existing EMR systems to conduct real-time screening and identification of fall events within minutes. For instance, upon discharge, health care professionals can use it to screen older patients with a history of falls to be recruited in posthospital fall prevention programs. After discharge, patients’ fall-related readmission can be tracked and identified timely by this strategy in the EMR system. To establish a more comprehensive surveillance procedure, supplementing nonmedical methods to collect fall incidents that do not present hospitals can be considered. Meanwhile, posthospital health care services, such as the Nurse and Allied Health Clinic (NAHC) for Falls, are designed specifically to prevent falls for patients. Especially for a resource-limited setting, such services can better target patients with a fall history.

The strengths of this study include using 12-year medical records from a territory-wide older population across multiple hospitals, which enhances the reliability and generalizability of the developed NLP algorithm. In addition, we compared the performance of independent and combined fall identification strategies based on *ICD* codes and clinical notes to determine the optimal method in practice. However, several limitations should be acknowledged. First, direct comparison of our NLP algorithm with existing rule-based and ML-based NLP algorithms, including transformer-based pretrained models, was not conducted in this study. Future research should compare the performance of these approaches on the same set of inpatient notes and evaluate their strengths and limitations. Second, a comprehensive analysis of false positives and negatives pattern could not be presented due to data sharing restrictions. Third, due to data availability, this study was limited by the inability to incorporate clinical notes from patients admitted to the ED to derive and compare NLP algorithms for different health care utilizations. Future studies are warranted to compare NLP methods generated from different settings and evaluate their contribution to fall identification. In addition, since the data in this study were drawn from 12-year medical records, it is uncertain whether the writing style of doctors or nurses changed over time. The lack of interrater reliability testing represents another limitation of this study. While the analysis of performance metrics for *ICD* codes would provide a more comprehensive comparison, this analysis was not conducted in this study and remains an important direction for future research. Finally, this study used a cohort developed for another study, which excluded older patients who had hospitalizations within the past year or were admitted from nursing homes, leading to a relatively healthier sample with lower fall risk. Investigations on the performance of our rule-based NLP algorithm in a more general older population are required.

### Conclusions

To conclude, this study has demonstrated that the rule-based NLP algorithm could efficiently and accurately detect falls in clinical notes from inpatient admission episodes with excellent performance. The combined identification strategy that incorporates the NLP algorithm and *ICD* codes could be applied to comprehensively capture fall episodes and fallers in future research of fall epidemiology, as well as in clinical practice to facilitate the identification of high-risk groups for fall interventions.

## Supplementary material

10.2196/65195 Multimedia Appendix 1 Regular expressions used to identify fall events.

10.2196/65195 Multimedia Appendix 2 Criteria for sure positive and sure negative fall indicators.

## Abbreviations

API: application programming interface
ED: emergency department
EMR: electronic medical record
HA: Hospital Authority
HADCL: Hospital Authority Data Collaboration Lab
*ICD*: *International Classification of Diseases*
*ICD-9-CM*: *International Classification of Diseases, Ninth Revision, Clinical Modification*
ML: machine learning
NAHC: Nurse and Allied Health Clinic
NLP: natural language processing
RegEx: Regular Expression
WHO: World Health Organization
