# Supplementary material for: Development and Validation of a Rule-Based Natural Language Processing Algorithm to Identify Falls in Inpatient Records of Older Adults: Retrospective Analysis
Source: JMIR Aging. 2025 Jul 8;8:e65195. doi: 10.2196/65195 (PMC12262146; doi:10.2196/65195)
Supplement: Multimedia Appendix 1 [file aging-v8-e65195-s001.docx]

**Section S1.**

Fall expression was searched by the RegEx of ['f(a|e)ll(s|en|er|ing)'] and ['s/f']. The latter expression was searched as doctors in Hong Kong used this short form in clinical notes to record slip and fall.
