# Supplementary material for: Development and Validation of a Rule-Based Natural Language Processing Algorithm to Identify Falls in Inpatient Records of Older Adults: Retrospective Analysis
Source: JMIR Aging. 2025 Jul 8;8:e65195. doi: 10.2196/65195 (PMC12262146; doi:10.2196/65195)
Supplement: Multimedia Appendix 2 [file aging-v8-e65195-s002.docx]

**Section S1.**

***The sure positive indicators***

1. Expressions indicating this inpatient admission was due to falls; (admit|(?<![a-zA-Z])adm|admission|admitted|presented|attended)( x(?![a-zA-Z])| for| after| due to| because of| with)?(?:\s+\w+){{0,{k}}}\s+(fall|fell|sf(?![a-zA-Z])|slip and fall|slip and fell|slipped and fall|slipped and fell|trip and fall|trip and fell|tripping and fall|tripping and fell|tripped and fall|tripped and fell)
2. Expressions indicating falls happened recently; (fall|fell|(?<![a-zA-Z])sf|slip and fall|slip and fell|slipped and fall|slipped and fell|trip and fall|trip and fell|tripping and fall|tripping and fell|tripped and fall|tripped and fell)(?:\s+\w+){{0,{k}}}\s+(day of admission|morning of admission|afternoon of admission|today|this morning|this am(?![a-zA-Z])|this afternoon|this pm(?![a-zA-Z])|tonight|yesterday|last night|monday|mon(?![a-zA-Z])|tuesday|tue(?![a-zA-Z])|wednesday|wed(?![a-zA-Z])|thursday|thu(?![a-zA-Z])|friday|fri(?![a-zA-Z])|saturday|sat(?![a-zA-Z])|sunday|sun(?![a-zA-Z])|last monday|last mon(?![a-zA-Z])|last tuesday|last tue(?![a-zA-Z])|last wednesday|last wed(?![a-zA-Z])|last thursday|last thu(?![a-zA-Z])|last friday|last fri(?![a-zA-Z])|last saturday|last sat(?![a-zA-Z])|last sunday|last sun(?![a-zA-Z])|this week|last week|this month|last month|ago|day ago|days ago|week ago|weeks ago|month ago|months ago|before|day before|days before|week before|weeks before|month before|months before|prior|day prior|days prior|week prior|weeks prior|month prior|months prior)
3. Expressions indicating fall was one of the admission diagnoses. ((?<![a-zA-Z])co|complained|complained of|(?<![a-zA-Z])hpi|(?<![a-zA-Z])imp|impression|progress)(?:\s+\w+){{0,{k}}}\s+(fall|fell|sf(?![a-zA-Z])|slip and fall|slip and fell|slipped and fall|slipped and fell|trip and fall|trip and fell|tripping and fall|tripping and fell|tripped and fall|tripped and fell)

***The sure negative indicators***

1. Expressions indicating denied falls; (deny|denies|denied|(?<![a-zA-Z])no|(?<![a-zA-Z])not|(?<![a-zA-Z])nor|without)(?:\s+\w+){{0,{k}}}\s+(fall|fell|sf(?![a-zA-Z])|slip and fall|slip and fell|slipped and fall|slipped and fell|trip and fall|trip and fell|tripping and fall|tripping and fell|tripped and fall|tripped and fell|history of fall|hx of fall)
2. Expressions indicating fall was caused by specific external reasons; (?<![a-zA-Z])hit by(?:\s+\w+){{0,{k}}}\s+(fall|fell|sf(?![a-zA-Z])|slip and fall|slip and fell|slipped and fall|slipped and fell|trip and fall|trip and fell|tripping and fall|tripping and fell|tripped and fall|tripped and fell)
3. Expressions indicating inpatient falls; (fall|fell|(?<![a-zA-Z])sf|slip and fall|slip and fell|slipped and fall|slipped and fell|trip and fall|trip and fell|tripping and fall|tripping and fell|tripped and fall|tripped and fell) in ward
4. Expressions indicating fall risk, assessment, or prevention; (risk|risks) of( future| recurrent| repeated| frequent)? (fall|falls); (fall|falls) (risk|assessment|precaution|prevention); (precaution for|prevent) (fall|falls)
5. Expressions indicating fall tendency; (easy|easily|near|nearly|tend to|tendency to|tendency of|afraid of|fear of) (fall|fell|sf(?![a-zA-Z])|slip and fall|slip and fell|slipped and fall|slipped and fell|trip and fall|trip and fell|tripping and fall|tripping and fell|tripped and fall|tripped and fell)
6. Expressions indicating the term ‘fall’ was used in situations irrelevant to falls; (fall|falls|fell|falling) asleep; buffallo|fallopian|pitfall; ((?<![a-zA-Z])cea initial|(?<![a-zA-Z])esr|paradoxical|platelet|(?<![a-zA-Z])bp|blood pressure|rise to and|stone|stone which|window) (fall|fell); (fallen|falling|good falling) (vase|object|psa|tent|trend); ((?<![a-zA-Z])dr (sf(?![a-zA-Z]))
7. Expressions indicating the previous admission was due to falls. (recent|recently|previous|previously|last|lastly|last time|just|been) (admit|adm|admission|admitted|presented|attended)( x(?![a-zA-Z])| for| after| due to| because of| with)?(?:\s+\w+){{0,{k}}}\s+(fall|fell|sf(?![a-zA-Z])|slip and fall|slip and fell|slipped and fall|slipped and fell|trip and fall|trip and fell|tripping and fall|tripping and fell|tripped and fall|tripped and fell); (admit|(?<![a-zA-Z])adm|admission|admitted|presented|attended) (recent|recently|previous|previously|last|lastly|last time)( x(?![a-zA-Z])| for| after| due to| because of| with)?(?:\s+\w+){{0,{k}}}\s+(fall|fell|sf(?![a-zA-Z])|slip and fall|slip and fell|slipped and fall|slipped and fell|trip and fall|trip and fell|tripping and fall|tripping and fell|tripped and fall|tripped and fell); (admit|(?<![a-zA-Z])adm|admission|admitted|presented|attended)( x(?![a-zA-Z])| for| after| due to| because of| with)?(?:\s+\w+){{0,{k}}}\s+(fall|fell|sf(?![a-zA-Z])|slip and fall|slip and fell|slipped and fall|slipped and fell|trip and fall|trip and fell|tripping and fall|tripping and fell|tripped and fall|tripped and fell) (recent|recently|previous|previously|last|lastly|last time)
